# Dr. Edward Barnwell Bangasser and Dr. Sigismund Edward Inda

**Published:** 1917-08

**Authors:** 


**Dr. Edward Barnwell Bangasser,** Buffalo 1913 and **Dr. Sigismund Edward Inda,** Buffalo 1913, were killed in an automobile accident, July 18. They were traveling together to

New York and, near Dayton, owing to the condition of the roads, their auto skidded into a ditch. Both were dead when found, shortly afterward.

